# Novel drug-target interactions via link prediction and network embedding

**DOI:** 10.1186/s12859-022-04650-w

**Published:** 2022-04-04

**Authors:** E. Amiri Souri, R. Laddach, S. N. Karagiannis, L. G. Papageorgiou, S. Tsoka

**Affiliations:** 1 Department of Informatics, Faculty of Natural, Mathematical and Engineering Sciences, King’s College London, Bush House, London, WC2B 4BG UK; 2 St. John’s Institute of Dermatology, School of Basic and Medical Biosciences, King’s College London, Guy’s Hospital, London, SE1 9RT UK; 3 Breast Cancer Now Research Unit, School of Cancer and Pharmaceutical Sciences, King’s College London, Guy’s Cancer Centre, London, SE1 9RT UK; 4 Centre for Process Systems Engineering, Department of Chemical Engineering, University College London, Torrington Place, London, WC1E 7JE UK

**Keywords:** Machine learning, Graph-embedding, Drug repurposing, Link prediction

## Abstract

**Background:**

As many interactions between the chemical and genomic space remain undiscovered, computational methods able to identify potential drug-target interactions (DTIs) are employed to accelerate drug discovery and reduce the required cost. Predicting new DTIs can leverage drug repurposing by identifying new targets for approved drugs. However, developing an accurate computational framework that can efficiently incorporate chemical and genomic spaces remains extremely demanding. A key issue is that most DTI predictions suffer from the lack of experimentally validated negative interactions or limited availability of target 3D structures.

**Results:**

We report DT2Vec, a pipeline for DTI prediction based on graph embedding and gradient boosted tree classification. It maps drug-drug and protein–protein similarity networks to low-dimensional features and the DTI prediction is formulated as binary classification based on a strategy of concatenating the drug and target embedding vectors as input features. DT2Vec was compared with three top-performing graph similarity-based algorithms on a standard benchmark dataset and achieved competitive results. In order to explore credible novel DTIs, the model was applied to data from the ChEMBL repository that contain experimentally validated positive and negative interactions which yield a strong predictive model. Then, the developed model was applied to all possible unknown DTIs to predict new interactions. The applicability of DT2Vec as an effective method for drug repurposing is discussed through case studies and evaluation of some novel DTI predictions is undertaken using molecular docking.

**Conclusions:**

The proposed method was able to integrate and map chemical and genomic space into low-dimensional dense vectors and showed promising results in predicting novel DTIs.

**Supplementary Information:**

The online version contains supplementary material available at 10.1186/s12859-022-04650-w.

## Background

Discovering a new drug is a high-risk, time-consuming, and expensive process [1], a process that typically takes more than 15 years, costs $2.6 billion and is limited by less than 10% success rates [2, 3]. Therefore, there is strong interest to develop new efficient methods able to discover previously unknown activities of existing drugs to uncover new medical purposes outside the original scope, a process known as *drug repositioning* or *repurposing* [4]. Drug repurposing is a promising alternative to traditional drug discovery approaches, offering a shorter route to clinical development, bypassing several stages of clinical development [5] which have already been completed for the original target and reducing major risks, expense and time by several years [6]. The first major step in repositioning a drug is identifying possible potential target proteins by predicting valid DTIs [7].

Due to expensive and time-consuming laboratory experiments, limited availability of physical resources, and the complexity of integrating chemical and genomic spaces, just a small number of DTIs are experimentally validated. Therefore, accurate prediction of DTIs at large scale remains a challenge [8]. Recently, computational prediction methods based on machine learning (ML) principles have received increasing attention [9] because of their ability to integrate different types of biological data and analyse large numbers of possible interactions efficiently, leading to faster and cheaper assessments [10]. There is an urgent need for sophisticated computational modelling approaches [11] to limit the number of potential interactions which can be reasonably verifiable by in vitro screening [12].

Traditional computational methods in DTI prediction are mainly categorised in two types of strategies: molecular docking simulations and ligand-based approaches [13]. However, the applicability of docking simulations is limited by the availability of 3D crystal structures of target proteins which is still unknown for the majority of membrane proteins especially G-protein-coupled receptors (GPCR) [6]. Ligand-based approaches also suffer from low prediction rates when the number of known binding ligands is small [14]. To avoid the limitations of traditional methods, several ML-based models have been developed which achieved considerable success by translating large scale chemogenomics data to a set of features and extracting latent patterns in DTIs [15]. The most popular group of ML methods for DTI prediction is similarity methods which incorporate target-target and drug-drug similarity metrics. These rely on a key underlying assumption that similar drugs may tend to target similar proteins and vice versa [6]. Similarity-based approaches have several advantages which include the ability to connect chemical and genomic spaces and the availability of well-defined similarity measures between the chemical structure of drugs and genomic sequences [16]. By integrating multiple types of similarities into a heterogeneous network, determining new DTIs can be formulated as a link prediction problem in graph analysis [8].

Graph embedding algorithms are popular methods recently used in graph analytics to represent graph structural properties as a set of low dimensional vectors [17] which can be introduced into ML models as input features. The use of embedding methods to infer different biological interactions such as drug-drug [18], protein–protein [19], and drug-target [10] outperforms current state-of-the-art methods [18]. The “2vec” (short for “to vector”) models like “graph2vec”, “node2vec”, etc. are an important category of embedding algorithms inspired by the “word2vec model” [20] a popular word embedding algorithm in natural language processing, which used neural networks to learn word vectors from sentences [20]. The benefits conferred by representing DTI as a vector stem from its capability to incorporate heterogeneous chemical and genomic data into a unified space, in addition to the fact that different ML algorithms can handle numerical input features well. Researchers in recent years have developed different embedding methods for predicting DTIs, such as TriModel [21], DTI-HeNE [9] and DTiGEMS+ [10] based on drug chemical structure and protein sequence to build similarity networks (details in Additional file 1: Related work). Another embedding-based method for DTI prediction has looked into integrating multi-molecular associations such as protein, drug, disease, lncRNA, and miRNA from multiple databases into a heterogeneous network [22]. Besides embedding methods, a wide variety of computational algorithms have been proposed and summarized recently [6, 10, 23].

Although these models achieved promising performance, the lack of experimentally validated negative interactions is a common limitation of almost all supervised learning methods [8]. To this end, usually non-interacting drug-target pairs are assumed as negative samples. This affects the efficiency of developed models in real-life applications because it could include some positive interactions that have not been tested yet [21]. Moreover, as discussed previously, since the number of known DTIs is considerably smaller than unknown drug-target pairs (labelled as negative samples), it leads to imbalanced classification and skewed results. Therefore, selecting realistic negative interactions was highlighted as one of the important tasks in future developments of DTI prediction [24].

We have previously reported mathematical optimization as means of predicting the affinity of DTI [25, 26]. In this paper, we developed a computational framework that employs data from multiple DTI sources and formulates the problem of deriving new DTIs as link prediction. We used two datasets separately to evaluate and extract novel DTIs; the Golden Standard and ChEMBL datasets. The link prediction methodology employs the following stages: (1) creation of a drug-drug similarity network (2) creation of a protein–protein similarity network, (3) feature extraction using graph embedding of networks in (1) and (2) via node2vec, and defining interactions by concatenating pairs of drug and target features, (4) a classification scheme that employs the embedding features and gradient boosted trees to predict new DTIs. Method performance is based on an external validation metric of the classification model, evaluation of prediction is assessed via molecular docking of drugs predicted to bind the protein target and different case studies of newly predicted DTIs are discussed.

## Materials and methods

**Fig. 1:**
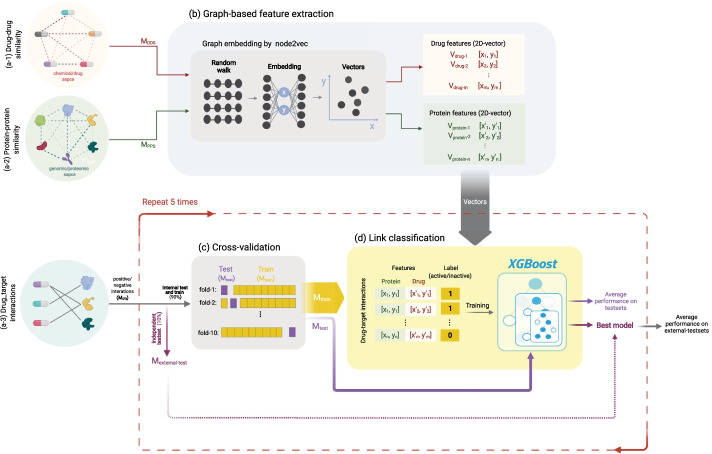
DT2Vec pipeline. **a1**, **a2** Drug-drug (DDS) and protein–protein (PPS) similarity graphs based on similarity used as input of embedding method to generate vectors. **a3** Graph of DTIs. **b** Graph-embedding developed by node2vec to map nodes (in DDS and PPS) to vectors (in this figure drugs and target mapped to 2D-vector, x and y). **c** Known DTIs (positive and negative) were divided into 10% independent dataset (external testset) and 90% internal test and train (tenfold cross-validation). **d** Drug and protein vectors were concatenated and labeled as positive (1) or negative (0) and an XGBoost model was trained on the cross-validation datasets. The best model over the tenfold cross-validation on the internal testset was selected and applied on the external testset. The XGBoost model in **c**, **d** was repeated 5 times and the average performance of internal and external testsets was reported

The overall computational framework is shown in Fig. 1 and includes data pre-processing, implementation of the proposed methodology and evaluation. As described below, first the performance of our approach is compared to similar methods from the literature on a standard benchmark dataset, and then a dataset containing experimentally verified positive and negative interactions was collected to detect more realistic DTIs and drug repurposing.

### Standard benchmark dataset

A *“Golden-standard dataset”* was introduced by Yamanishi et al. [27, 28] and has been used previously as a reference for predicting DTIs and comparing the performance of different models [1, 14, 21, 23, 29]. It consists of a binary drug-target edge-list M_DTI_ (Fig. 1: a3) and two similarity matrices in chemical and genomic spaces M_DDS_ (Fig. 1: a1) and M_PPS_ (Fig. 1: a2) respectively. Since experimentally validated negative interactions are not available, interactions between all possible drug-target pairs where a known interaction does not exist, were considered as the set of negative interactions in most studies [21, 27, 30]. Details of the “*Golden-standard dataset*” are given in Table 1a and Additional file 2.

**Table 1:** Dataset details (a) Golden-standard dataset (b) ChEMBL-based dataset

(a) Golden_standarad_dataset	# Drug	# Target	# Negative/unknown DTI	# Positive interaction	Class ratio
Enzyme (E)	445	664	292,554	2926	0.01
Ion channel (IC)	210	204	41,364	1476	0.04
G-protein-coupled receptors (GPCR)	223	95	20,550	635	0.03
Nuclear receptor (NR)	54	26	1314	90	0.07
Total	791	989	777,172	5127	0.01

(b) ChEMBL_dataset	# Drug	# Target	# Real negative DTI^†^	# Positive interaction*^, †^	Class ratio
CheMBL	548	556	2057	1721	0.84
	# Weak DTI**	# Unknown DTI^‡^			
	532	300,378			

^*^pChEMBL value ≥ 5.5, **pChEMBL value > 0, ^**†**^Development-dataset, ^**‡**^Experimental-dataset

In order to obtain a balanced dataset of positive and negative interactions, in most studies unknown interactions were selected randomly and labelled negative [6, 10, 21]. However, this set of randomly selected cases may include some real positive interactions that are yet unknown, which may lead to artefacts in DTI prediction [1, 23]. Therefore, in this case, the Recall metric (i.e. true positives/(true positives + false negatives)) can better reflect the performance of the models, as it is calculated on validated positive labels. In detecting new DTIs, false-positive predictions (i.e. negative interactions that were predicted to be positive) may be reported as newly discovered interactions [10, 14, 21, 28], thereby creating problems in distinguishing between false-positives caused by model error and newly discovered interactions. Therefore, although the Golden-standard dataset is convenient as a common benchmark dataset for comparing the performance of different developed models, its limitation is that it only contains experimentally validated positive interactions which are not suitable for training a realistic DTI prediction model [15]. Screening reliable true negative interactions was recently highlighted as one of the critical steps in improving the prediction accuracy of developed methods [24, 29] and this point is better addressed through the use of the dataset described below.

### A realistic dataset for drug repurposing extracted from the ChEMBL database

To address these limitations, a DTI dataset was collected from the ChEMBL repository [31] that contains experimentally validated negative and positive interactions. In literature, an activity threshold of pChEMBL of 5.0 is typically used to label an interaction as active. In chemical assay experiments, the acceptable model results should be higher than 10 μM affinity (or pChEMBL = 5) [32]. To ensure that the positive interactions are strong and consequently offer more accurate prediction results, activity greater than 5.5 was chosen [33]. Positive (pChEMBL ≥ 5.5) and negative (labelled as inactive interactions in ChEMBL repository) interactions form a binary edge-list M_DTI_ (Fig. 1: a3).

Drug similarity measures were calculated through MACCS [34] based on structural information which can codify 166 structural fingerprints in bit positions. Then, we measured the similarity between drug pairs using the Tanimoto coefficient in the range of 0 to 1 [35]. Open Babel [36] in Python 3.7.3 was used to generate the drug-drug similarity network M_DDS_ (Fig. 1: a1) [37]. The protein similarity network, M_PPS_ (Fig. 1: a2), was computed using sequence alignment [38], implemented through the parallelised version of protein similarity calculation using the “protr” package in R 4.0.2 [39]. Additional file 3: Figure S1 and Additional file 2 describe steps performed to collect the ChEMBL-based dataset. Known positive and negative DTIs comprise the *development-dataset* (Table 1), a set of interactions used to build the ML model. All possible drug-target pairs with no known (active or inactive) interactions in ChEMBL (Table 1) were defined as the *‘experimental-dataset’* and were used for predicting interactions and performing drug repurposing.

### Development and evaluation of the DT2Vec model

We report DT2Vec, an ML method for drug-target interaction prediction, trained on features extracted using graph embeddings. DT2Vec was implemented and evaluated on the Golden-standard dataset as well as experimentally validated datasets extracted from ChEMBL. The first dataset was used as a benchmark to validate the performance of the developed model through comparison with three state-of-the-art open-source chemogenomic algorithms that employed the Golden-standard database, namely DNILMF [40], DT-Hybrid [41], DDR [14]. These methods are based on graph similarity algorithms and are reported as top-performing methods in DTI prediction [1, 10]. Details of these methods are in Additional file 1: Related work. After benchmarking, the DT2Vec model was implemented on the ChEMBL based dataset which included first training the model on known positive and negative interactions and then applying the model on unknown DTIs to detect novel interactions.

#### Graph-based feature generation using node2vec

The performance of ML algorithms is highly dependent on choosing a set of informative and discriminative features. Many ML methods benefit from semantically meaningful features, automatically extracted from highly structured objects like graphs which not only reduce the manual feature engineering effort but also enhance the predictive capability of the model. As shown in Fig. 1b, in order to extract features, node2vec [42], a semi-supervised feature learning algorithm was applied to the weighted graph of drug-drug and protein–protein similarities separately to embed drug and target nodes into a continuous vector space with n-dimensions (as an example, in Fig. 1b the embedded vectors, V_drug_ and V_protein_ are two-dimensional). Based on recent ML research, node2vec outperforms other existing state-of-the-art methods in node embedding [17, 42, 43]. Recently, node2vec showed promising results on DTI prediction by mapping drug, protein, disease, lncRNA and miRNA association networks to vectors [44]. We used node2vec implemented in Python 2 using the source code available in GitHub [43]. DTIs were defined by concatenating the embeddings of the drug and protein similarity networks and then were used as input features for an ML classifier. The drug-drug and protein–protein similarity networks (Fig. 1a, b) were clustered using Louvain [45] to obtain a topological characterisation of the structure of the networks and networkX [46] was used to visualize the networks. Then the drug and target embedded vectors are visualized based on two principal components using PCA [47] to illustrate how the embedding vectors represent the communities in the networks.

#### Data-partitioning and cross-validation

There are several strategies that can be used in validating DTI prediction models [48]. Cross-validation (CV) schemes are a robust strategy to estimate how a model generalizes, whereby data is split multiple times to increase the variation in the training and testing data. The developing processes employed internal and external testing whereby the drug-target edge lists, M_DTI_, were split into 90% internal training (80% train, 10% internal-test) and 10% external testset, repeated 5-times tenfold CV (Fig. 1c). The best model was selected based on the internal test set and assessed on the external testset which is blind to the process of developing the model, to obtain a more realistic representation of generalised performance [49]. DT2Vec was trained and tested on the Golden-standard (positive and randomly selected unknown interactions) and ChEMBL (positive interactions with pChEMBL ≥ 5.5 and experimentally validated negative interactions, named *‘development_dataset’*) datasets. After validating the performance of the method using CV, the final model is built on the whole data. Then the final model on the ChEMBL dataset was applied to the unknown ChEMBL interactions (named ‘*experimental-dataset*’) to detect novel DTIs. Details are shown in Additional file 4: Fig. S2.

#### Machine learning-based link classification

DTIs were represented as a 2n-dimension vector (n = 100, Fig. 1d shows n = 2 as an example) by concatenating the drug and target embedding features and labelled as “active” or “inactive” as described previously [17]. The DTI prediction problem was formulated as a binary classification problem built on XGBoost [8] (Fig. 1d). XGBoost is a stochastic gradient boosting algorithm which combines weak ensemble decision trees and was selected due to its high speed, accuracy, and ability to handle imbalanced datasets [4]. Moreover, by taking advantage of XGBoost returning the prediction probability score, we were able to rank DTIs based on the confidence score that the model provides. Grid-search was performed on training set samples within each cross-validation fold to find the best set of hyperparameters. The model was implemented in Python 3.7.3, using XGBoost 0.90 with hyperparameters of maximum tree depth = 4, subsample ratio = 1, minimum child weight = 2, and learning-gamma rate = 0.8. To evaluate the performance of the model, the average Precision, Recall, and f_β_-score across all cross-validation sets are calculated.

### Extracting new DTIs

In order to demonstrate the use of the DT2Vec for drug repurposing, novel DTIs were predicted from a dataset of all drug-target pairs in ChEMBL where interaction is not known (*experimental-dataset*). After benchmarking the model through cross-validation, DT2Vec was built on known DTIs in the *development-dataset*, before being applied to the *experimental-dataset*. Selected newly predicted DTIs by our method were assessed by performing docking. First, predicted DTIs were selected by two criterial: (1) a probability score by XGBoost ≥ 0.99%, and (2) DTIs having drugs in phase-4 clinical trials. The amino-acid sequence of protein targets of interest was used to obtain PDB structures for docking (https://www.rcsb.org/). Chains attributed to homo sapiens were used to calculate the similarity score based on sequence alignment using protr [39] with default settings in R 4.0.5. SwissDock [50] was used with default parameters to perform drug-protein docking.

## Results

### Implementation of DT2Vec model on the benchmark Golden standard dataset and comparison with other methods

We developed and evaluated our proposed embedding-based DTI model (DT2Vec) on the benchmark Golden-standard dataset (Table 1a) [27]. Node2vec was used on DDS and PPS to map them to 100-dimension vectors which reported as the best vector size for preserving network neighborhoods of nodes [10, 17, 42, 43]. To obtain a topological characterisation of the networks, the drug and protein similarity networks were clustered which consisted of 4 (with 360, 203,181, and 47 drug members) and 7 (with 484, 204, 120, 95, 42, 26, and 18 protein members) drug and protein communities respectively as shown Additional file 5: Fig. S3 a-1, a-3. Additional file 5: Figure S3 a-2, a-4 show PCA of drugs and targets based on embedded vectors as features and colours indicating cluster membership in DDS and PPS networks. It was observed (Additional file 55: Fig. S3a) that the embedded vectors based on the Golden-standard dataset can represent the topological features of networks well. In Additional file 6: Fig. S4a, the PCA of embedded vectors in the protein target similarity dataset is shown, coloured according to protein type (i.e. enzymes, GPC receptors, nuclear receptors, and ion channels).

To create DTI labels in the Golden-standard dataset, as known negative samples are not available, unknown interactions were randomly selected to create negatively labelled samples. This assumption leads to unreliable false-positive predictions (and therefore Precision, i.e. true-positives/(true-positives + false-positives)), so the f_2_-score, which weighs Recall higher than Precision, was deemed suitable. However, since a trade-off between Precision and Recall exists, the goal of the model should be high Recall without sacrificing Precision. The performance was measured based on tenfold cross-validation which was repeated five times and compared with three methods DNILMF [40], DT-Hybrid [41], DDR [14]. The DT2Vec model achieved f2-score, Recall, Precision average of 91.69% (1.5), 92.63% (0.82), 88.13% (0.5) which was better than DNILMF with 87.92% (1.4), 87.84% (1.63), 88.27% (1.84), DT-Hybrid with 72.7% (1.2), 70.76% (1.55), 81.72% (0.95) and DDR with 89.87% (1.25), 89.83% (1.4), 90.08% (1.07) respectively.

### Development of DT2Vec on ChEMBL interactions

A DTI dataset was collected from ChEMBL containing experimentally validated negative and positive interactions in order to offer a more realistic interaction set (Table 1b). ChEMBL DDS and PPS networks were clustered to 4 (with 193, 150, 105, and 100 drugs) and 5 (with 304, 153, 86, 8, and 5 proteins) communities respectively (Additional file 5: Fig. S3 b-1, b-3). Additional file 5: Figure S3 b-2, b-4 shows PCA of drugs and targets using the embedding vectors colored based on the cluster membership, showing that node2vec vectors can represent the topological properties of the original networks well. For reference, Additional file 6: Fig. S4b shows PCA of protein target embedding vectors colored according to protein type.

Interactions in the ChEMBL dataset were divided into (1) a *development-dataset* comprising 2057 negative and 1721 positive (pChEMBL value ≥ 5.5) interactions, and were used to train and test DT2Vec, (2) an *experimental-datase*t, comprising all unknown interactions (a total of 300,378), which were used to predict and extract novel interactions and evaluate performance on independent datasets. In contrast to the benchmark Golden-standard dataset, in ChEMBL data the DT2Vec model was trained with experimentally verified negative interactions, therefore false-positive predictions and the Precision metric are more realistic, and the f_1_-score (which weights Precision and Recall equally) was used. The average performance through 5 times tenfold CV on the external test sets was calculated and the model achieved high Precision of 92.79% (0.02) showing low false-positive predictions and indicating that the model can accurately predict novel DTIs. The model also demonstrated promising results on Recall, f1-score, AUPR and AUC with 92.88% (0.02), 92.82% (0.01), 89.42% (0.02), and 94.09% (0.01) respectively (Additional file 7: Fig. S5 shows ROC plot across 5 runs).

#### Extracting and evaluating DTIs

**Fig. 2:**
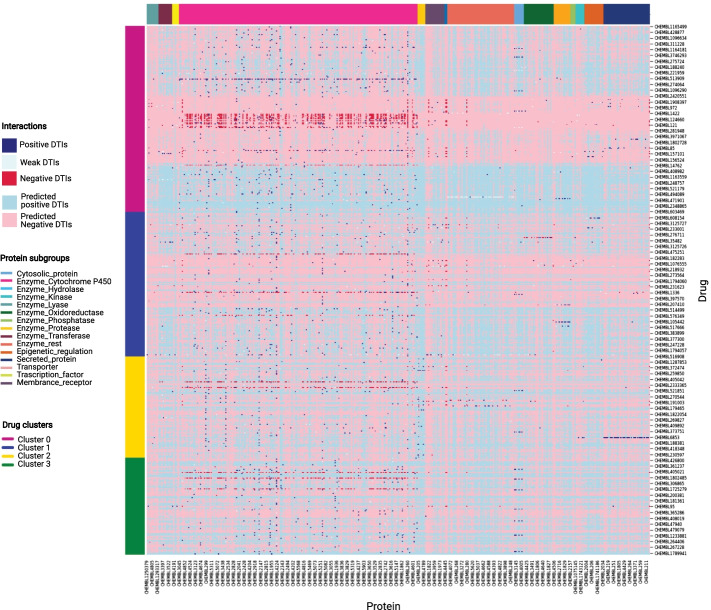
Heatmap to show the mapping of known DTI interactions (dark blue, dark red) to predicted interactions (light blue, pink), labeled based on protein subgroups (columns) and drug network clusters (rows)

In order to demonstrate the use of the DT2Vec for drug repurposing, novel DTIs were predicted from a dataset of all drug-target pairs in ChEMBL where interaction is not known (*experimental-dataset*). Figure 2 shows DTIs as a heatmap where all *known* drug-target interactions (dark blue for positive or red for negative) and *predicted* DTIs (light blue for positive or red for negative) are mapped, with proteins in columns coloured according to subgroup and drugs in rows coloured by chemical similarity. By comparing known and predicted DTIs in Fig. 2, we illustrate that prediction via DT2Vec can extend beyond the ‘similar drug for similar target’ principle, which has traditionally been the basis of various drug repurposing efforts.

**Fig. 3:**
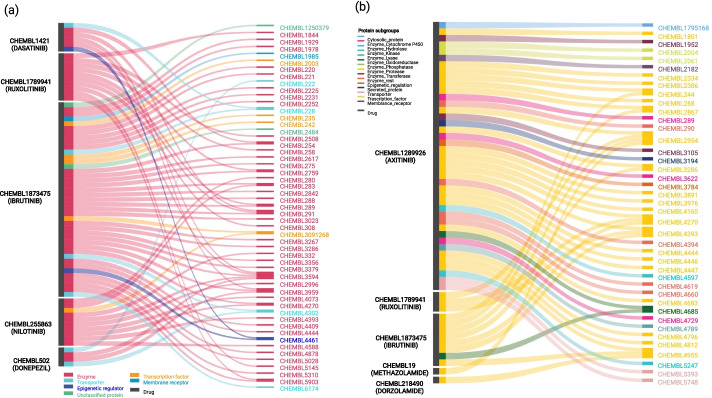
Illustration of key interactions between drugs (left) and protein targets (right). **a** Predicted highly-ranked positive interactions for phase-4 drugs with interactions colored based on protein type. **b** Phase-4 drugs repurposed for protein without any approved drugs based on the selected dataset. Colours show the type of protein target

To further illustrate the nature of predicted DTIs via DT2vec, the top novel positive interactions where a drug has been approved at phase-4 clinical trial are shown (Fig. 3a). In the *development-dataset* (known DTIs), 387 (out of 556) proteins have known positive interactions and only 162 can be associated with phase-4 drugs (394 without any approved drugs). Figure 3b shows the top predicted positive interactions for proteins without any phase-4 drugs in this dataset, which represent cases where repurposing may be highly promising.

**Fig. 4:**
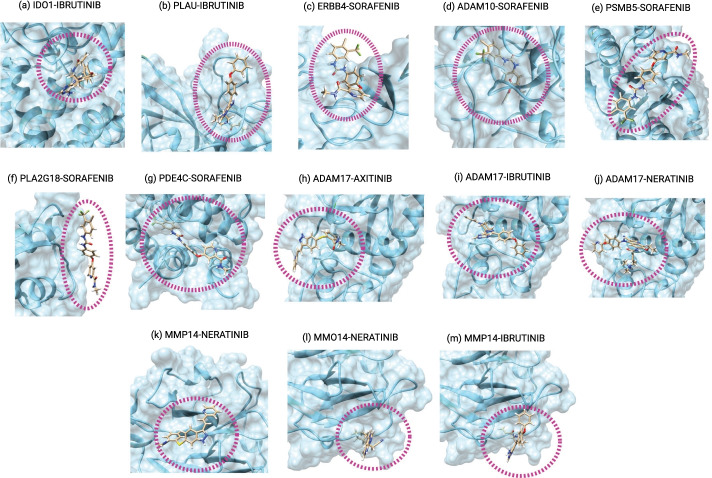
Drug-target docking by SwissDock for new DTIs, showing binding with the lowest deltaG

Drug-target docking was used to investigate the validity of some newly predicted DTIs and shed light on the relevant molecular interaction. Figure 4 and Additional file 8: Fig. S6 show docking results and visualised with UCSF Chimera for new predicted DTIs. The free energy of binding (deltaG) for the first 10 docking groups is shown in Additional file 8: Fig. S6a. In general, negative deltaG indicates favourable binding of drg to the respective protein. Among 556 target proteins in the ChEMBL dataset, 77 proteins cannot be associated to a known 3D structure, indicating challenging cases where ML-based DTI prediction can be particularly advantageous for drug discovery. Additional file 10: Table S1 shows predicted phase-4 drugs (approved drugs) for the set of proteins with no known 3D structure. The next section discusses the evaluation of drug-target interactions in more detail.

## Discussion

We report a novel methodology for drug repurposing based on graph embedding and below we evaluate our method by focusing on case studies of newly predicted DTIs that we identified. In this section, we provide extensive discussion of novel DTIs that are associated with approved drugs with multiple highly positive new interactions, cancer-related targets [51], and proteins without 3D-structure data [6] where drug discovery is more challenging and therefore of particular interest.

As demonstrated in Fig. 3a, based on DT2Vec prediction, IBRUTINIB (CHEMBL1873475) targeted multiple proteins which are important in repurposing. IBRUTINIB is a known inhibitor of Bruton’s tyrosine kinase (BTK). By acting downstream of the B cell receptor (BCR), IBRUTINIB can block malignant B cell signalling and activation and lead to apoptosis [52, 53]. Based on the known interactions in *development-dataset*, IBRUTINIB can bind to eleven proteins (BTK, BLK, PSCTK4, BMX, EGFR, ERBB2, ERBB4, PTK4, CDHF12, ITK, and ERG). In predictions obtained by our method, it is indicated that it targets eight proteins that have no approved drugs based on our dataset (Fig. 3b). Emerging evidence shows that some of these genes, such as CHEMBL4685 (IDO1) and CHEMBL3286 (PLAU), are linked to cancer development. Specifically, IDO1, a metabolic enzyme involved in tryptophan metabolism and an interferon-induced checkpoint molecule associated with immune suppression, has been linked to many types of cancer, such as acute myeloid leukaemia, ovarian cancer or colorectal cancer. It is indicated that IDO1 is part of the malignant transformation process, helping malignant cells escape eradication by the immune system. Inhibiting IDO1 could increase the effect of chemotherapy as well as other immunotherapeutic protocols [54–57]. In the case of PLAU, elevated expression levels are found to be correlated with malignancy, it is more commonly associated with cancer progression than the tissue plasminogen activator (tPA) [58] and inhibitors to this target have been sought as anticancer agents. It is noted that clinical evaluation of these agents is hampered by incompatibilities between human and murine biology. Moreover, urokinase is used by normal cells for tissue remodelling and vessel growth, which necessitates distinguishing cancer-associated urokinase features for specific targeting [59, 60]. Molecular docking was used to validate the interactions between IBRUTINIB and these two protein targets, showing favourable interactions where IBRUTINIB can bind to one region in PLAU and three regions in IDO1 (Additional file 8: Fig. S6 ab,1-2).

In another example, among phase-4 clinical trial drugs in the *development-dataset,* SORAFENIB (CHEMBL1336) was linked to having one of the highest positive interactions. SORAFENIB is a kinase inhibitor approved for treating patients with inoperable liver cancer [61] and metastatic renal cell carcinoma. DT2Vec predicted five new targets for this drug (probability score ≥ 0.99%): ERBB4 (CHEMBL3009), ADAM10 (CHEMBL5028), PSMB5 (CHEMBL4662), PLA2G1B (CHEMBL4426) and PDE4C (CHEMBL291). Specifically, ERBB4 has been recently found to be expressed in several tumours and tumour cell lines and its inhibition can slow tumour growth [62]. ADAM10 is likely to be involved in breast cancer progression, especially in the basal subtype [63]. PSMB5 is associated with proliferation and drug resistance in triple-negative breast cancer [64]. Recent studies point to a relationship between PLA2G1B [65] and PDE4C [66] with cancer. The docking results validating the interactions are shown in Fig. 4 (details in Additional file 7: Fig. S5 a,b3-7). Therefore, there is considerable evidence to support repurposing SORAFENIB to these new targets.

Immune evasion in cancer is an unsolved problem affecting the efficacy of immunotherapies and decreasing patient survival [67]. We selected the cases of ADAM17 and MMP14 targets to illustrate the potential of our methodology, as they have been implicated in tumour evasion through metalloproteinase function and catalysis of cleavage of endogenous MHC class I-related chain molecule (MIC) A and B [68]. NK cells recognise and become activated by interacting with MIC via the NKG2D receptor. The soluble form of MIC (sMIC) can also bind to NKG2D, which is internalised and subsequently reduces NK anti-tumoural functions [69, 70]. Two PDB entries with the same similarity score have been found for ADAM17 (2I47 and 3G42); 2I47 was selected due to better resolution and chain A was used for docking [71]. Three PDB entries were identified for MMP14 with the same similarity score (3C7X, 6CLZ, 6CM1) and 3C7X was selected [72]. There were 17 drugs predicted to interact with ADAM17 (Additional file 11: Table S2a) and 28 for MMP14 (Additional file 11: Table S2b). The DDS was used to assess the similarity between structures predicted to bind to each of the targets. This was in order to identify any bias towards a common core structure shared between drugs, which could indicate that drugs were identified only based on structural similarity. We note that some drugs predicted by the method are structurally different according to fingerprint similarity, which increases the number of potential therapeutic options (Additional file 9: Fig. S7). CHEMBL1289926, CHEMBL1873475, and CHEMBL18002 were identified as having the highest probability of interaction with ADAM17. Similarly, CHEMBL1289926, CHEMBL1873475 and CHEMBL1789941 were predicted to have positive interaction with MMP14. The docking results of these new interactions were shown in Fig. 4 (details in Additional file 8: Fig. S6 a,b8-13). The deltaG for the first 10 groups of molecules indicates favourable binding.

Other interesting examples are proteins without known 3D-structure for which drug repurposing can have a significant impact. For example, EPHA6 (CHEMBL4526), plays an important role in the formation of breast cancer and poses a new therapeutic target for patients with ER-negative and HER2 positive [73]. Based on DT2Vec prediction, four approved drugs AXITINIB (CHEMBL1289926), IBRUTINIB (CHEMBL1873475), DORZOLAMIDE (CHEMBL218490), AFATINIB (CHEMBL1173655), and DONEPEZIL (CHEMBL502) can bind to EPHA6 with the probability score ≥ 0.99%. AXITINIB has been shown to offer promising results on inhibiting the growth of breast cancer in animal models [74], renal cell carcinoma in clinical trials [75] and several other tumour types [76]. As mentioned before, IBRUTINIB is known as a cancer growth inhibitor and inducer of apoptosis [52, 53]. AFATINIB is also approved as a treatment for lung cancer [77], breast cancer and other cancer types [78]. Finally, DORZOLAMIDE has shown antitumor activity which affects TXNIP-dependent tumour suppression pathways and also causes downregulation in the level of bcl-2 in cancer cells. A previous study also provided evidence for synergistic antitumor activities of DORZOLAMIDE and mitomycinC against Ehrlich ascites carcinoma tumour growth in vivo, and this might offer a potential combination to evaluate in future clinical studies [79]. It shows that the method was able to suggest new tumour inhibitor drugs for a protein of unknown 3D structure with a crucial role in cancer [80].

## Conclusion

In overview, drug repurposing is a promising avenue in drug discovery, supporting the discovery of new protein targets for an approved drug. The availability of large scale chemogenomic data, coupled with the efficiency of ML methodologies, can support drug discovery in a time-efficient and cost-effective manner. In this study, we present a machine learning pipeline that combines network embedding and gradient boosted tree classification, to cast a link prediction strategy for detecting new DTIs. The model was implemented and validated via mining two different drug-target datasets, and evaluations of DTIs included molecular docking simulations and reviewing the literature. A key advantage of our method is that it does not require a priori 3D structure information, and relies solely on drug chemical structures and protein sequences for proposing promising repurposing cases. We note that predicting new and previously unknown DTIs is not only important for drug repositioning purposes, but can also improve our understanding of drug side effects which are usually caused by unexpected interactions of drugs with off-target proteins.

## Supplementary Information

**Additional file 1: Related work**. Related methods from literature. Summary of different embedding and similarity based methods for DTI prediction.

**Additional file 2: Datasets**. Details about datasets used in this study are provided.

**Additional file 3: Figure S1**. Data collection procedure. (a) Workflow to collect data from the ChEMBL database, (b) Scatter plot of the pChEMBL value of collected DTIs on a boxplot.

**Additional file 4: Figure S2**. Datasets and data splitting. Details of data types and data splitting in cross-validation.

**Additional file 5: Figure S3**. Networks of drug and protein similarities. Topological representation of the DDS and PPS networks and PCA of drugs and targets based on embedded vectors.

**Additional file 6: Figure S4**. PCA plots. PCA of embedded vectors of proteins coloured according to protein type.

**Additional file 7: Figure S5**. ROC plots over the ten-fold five times cross-validation.

**Additional file 8: Figure S6**. DTIs through docking. Molecular docking performed via SwissDock for novel predicted DTIs. (a) deltaG for the first 10 groups of molecules clustered by conformers similarity. (b) Binding locations with the lowest deltaG and all groups of conformers.

**Additional file 9: Figure S7**. Drug similarity heatmaps. Drug-drug similarity for drugs predicted to interact with (a) ADAM17 and (b) MMP14.

**Additional file 10: Table S1**. Predicted phase-4 drugs for proteins of unknown 3D structure. List of potential phase-4 drugs (approved drugs) that can interact with unknown 3D structure proteins.

**Additional file 11: Table S2**. Predicted drugs for MMP14 and ADAM1. List of potential approved drugs that can target MMP14 and ADAM17.

## Data Availability

The datasets analysed during the current study are available in the ChEMBL repository, https://www.ebi.ac.uk/chembl/, and Yamanishi et al. article (https://doi.org/10.1093/bioinformatics/btn162) and its supplementary information files http://web.kuicr.kyoto-u.ac.jp/supp/yoshi/drugtarget/. The code is available at https://github.com/elmira-amiri/DT2Vec.

## Abbreviations

CV: Cross-validation
DDS: Drug-drug similarity
DTI: Drug target interaction
GPCR: G-protein-coupled receptors
ML: Machine learning
PCA: Principal component analysis
PPS: Protein–protein similarity
tPA: Tissue plasminogen activator
