# Supplementary material for: Novel drug-target interactions via link prediction and network embedding
Source: BMC Bioinformatics. 2022 Apr 4;23:121. doi: 10.1186/s12859-022-04650-w (PMC8978405; doi:10.1186/s12859-022-04650-w)
Supplement: Supplementary file 1 — Additional file 1: Related work. Related methods from literature. Summary of different embedding and similarity based methods for DTI prediction. [file 12859_2022_4650_MOESM1_ESM.docx]

## **Additional file 1: Related work**

**Embedding based methods:**

- **DTiGEMS+** [[1]](https://www.zotero.org/google-docs/?woYMeP): the method integrated multiple similarity matrices to calculate drug-drug (DDsim) and target-target (TTsim) similarity matrices and just kept the top strong similarities. The integrated DD sim, TT sim and DT interaction graphs (G1) fed into the node2vec. Then, to create G2 a new DDS (Md) and PPS (Mt) graph, cosine similarity is calculated and the weak similarities are removed from the graph. The features are extracted using G1 and G2 graphs based on path scores between each drug-target pair. Different supervised machine learning methods are used (Neural Networks, Random Forest, and Adaptive Boosting) to develop the DTI model. The Golden Standard Dataset was used to evaluate the performance of the proposed model.
- **TriModel** [[2]](https://www.zotero.org/google-docs/?oqgnlH):The model is based on biomedical knowledge bases in drugs and targets to generate a knowledge graph. An efficient model was trained to map the knowledge graph to three embedding vectors for each drug and target. A metric was defined to score interaction between possible drug target pairs. The performance of the model was benchmarked using the Golden Standard Dataset and a new KEGG based dataset.
- **DTI-HeNE** [[3]](https://www.zotero.org/google-docs/?qGL6Nx): A drug-target interaction prediction model based on a multi-staged embedding. The interactions formulated as a binary link-prediction and Drug-target embedding pairs were generated by integrating three varieties of networks (DTI network, drug homogeneous networks and target homogeneous networks). Drug/target nodes were generated by Bipartite Network Embedding (BiNE). Pathway information between these networks was used to integrate extracted features from the previous step and fed into Random Forest as input features to predict new DTIs.

**Graph similarity-based:**

- **DNILMF** [[4]](https://www.zotero.org/google-docs/?erGiBR): DNILMF is a matrix factorization on a dual-network to predict DTI which consists of four steps of creating kernel matrix, diffusing drug and target kernel matrix with drug structure and target sequence respectively, developing a predictive model. The results compared with NRLMF [[5]](https://www.zotero.org/google-docs/?YhmlOK). The model compared with NRLMF and EigenTrans based on the Golden Standard Dataset.
- **DT-Hybrid** [[6]](https://www.zotero.org/google-docs/?S9mRlx): Domain tuned-hybrid (DT-Hybrid) is a domain-based knowledge including drug and target similarity. DT-Hybrid is based on a recommendation method developed on bipartite network projection. The algorithm compared with two methods Hybrid [[7]](https://www.zotero.org/google-docs/?sycxgM) and NBI [[8]](https://www.zotero.org/google-docs/?SMAYzz) on the Golden Standard dataset and a database obtained from DrugBank.
- **DDR**[[9]](https://www.zotero.org/google-docs/?kkHOe6)**:** DDR used a nonlinear method to combine different similarities and path-category-based features were extracted from the heterogeneous graph used to build a DTI prediction model. The extracted graph based features were fed to a Random Forest, a supervised machine learning algorithm for predicting new DTIs . DDR performance was compared with COSINE [[10]](https://www.zotero.org/google-docs/?gDPYX2), NRLMF [[11]](https://www.zotero.org/google-docs/?jD0BLI), KRONRLS-MKL [[12]](https://www.zotero.org/google-docs/?pKlkw6) and BLM-NII [[13]](https://www.zotero.org/google-docs/?KQv2Fi) on the Golden Standard Dataset and DrugBank database (for drugs with FDA approval).

## **References**

[1. Thafar MA, Olayan RS, Ashoor H, Albaradei S, Bajic VB, Gao X, et al. DTiGEMS+: drug–target interaction prediction using graph embedding, graph mining, and similarity-based techniques. Journal of Cheminformatics. 2020;12:44.](https://www.zotero.org/google-docs/?o8UyjR)

[2. Mohamed SK, Nováček V, Nounu A. Discovering protein drug targets using knowledge graph embeddings. Bioinformatics. 2020;36:603–10.](https://www.zotero.org/google-docs/?o8UyjR)

[3. Yue Y, He S. DTI-HeNE: a novel method for drug-target interaction prediction based on heterogeneous network embedding. BMC Bioinformatics. 2021;22:418.](https://www.zotero.org/google-docs/?o8UyjR)

[4. Hao M, Bryant SH, Wang Y. Predicting drug-target interactions by dual-network integrated logistic matrix factorization. Sci Rep. 2017;7. doi:10.1038/srep40376.](https://www.zotero.org/google-docs/?o8UyjR)

[5. Liu Y, Wu M, Miao C, Zhao P, Li X-L. Neighborhood Regularized Logistic Matrix Factorization for Drug-Target Interaction Prediction. PLoS Comput Biol. 2016;12:e1004760.](https://www.zotero.org/google-docs/?o8UyjR)

[6. Alaimo S, Pulvirenti A, Giugno R, Ferro A. Drug–target interaction prediction through domain-tuned network-based inference. Bioinformatics. 2013;29:2004–8.](https://www.zotero.org/google-docs/?o8UyjR)

[7. Zhou T, Kuscsik Z, Liu J-G, Medo M, Wakeling JR, Zhang Y-C. Solving the apparent diversity-accuracy dilemma of recommender systems. PNAS. 2010;107:4511–5.](https://www.zotero.org/google-docs/?o8UyjR)

[8. Cheng F, Liu C, Jiang J, Lu W, Li W, Liu G, et al. Prediction of Drug-Target Interactions and Drug Repositioning via Network-Based Inference. PLOS Computational Biology. 2012;8:e1002503.](https://www.zotero.org/google-docs/?o8UyjR)

[9. Olayan RS, Ashoor H, Bajic VB. DDR: efficient computational method to predict drug–target interactions using graph mining and machine learning approaches. Bioinformatics. 2018;34:1164–73.](https://www.zotero.org/google-docs/?o8UyjR)

[10. Lim H, Gray P, Xie L, Poleksic A. Improved genome-scale multi-target virtual screening via a novel collaborative filtering approach to cold-start problem. Scientific Reports. 2016;6:38860.](https://www.zotero.org/google-docs/?o8UyjR)

[11. Liu Y, Wu M, Miao C, Zhao P, Li X-L. Neighborhood Regularized Logistic Matrix Factorization for Drug-Target Interaction Prediction. PLOS Computational Biology. 2016;12:e1004760.](https://www.zotero.org/google-docs/?o8UyjR)

[12. Nascimento ACA, Prudêncio RBC, Costa IG. A multiple kernel learning algorithm for drug-target interaction prediction. BMC Bioinformatics. 2016;17:46.](https://www.zotero.org/google-docs/?o8UyjR)

[13. Mei J-P, Kwoh C-K, Yang P, Li X-L, Zheng J. Drug–target interaction prediction by learning from local information and neighbors. Bioinformatics. 2013;29:238–45.](https://www.zotero.org/google-docs/?o8UyjR)
