# Supplementary material for: Novel drug-target interactions via link prediction and network embedding
Source: BMC Bioinformatics. 2022 Apr 4;23:121. doi: 10.1186/s12859-022-04650-w (PMC8978405; doi:10.1186/s12859-022-04650-w)
Supplement: Supplementary file 2 — Additional file 2: Datasets. Details about datasets used in this study are provided. [file 12859_2022_4650_MOESM2_ESM.docx]

## **Additional file 2**

Golden standard dataset, the most popular dataset in DTI predictions [1],[[2]](https://www.zotero.org/google-docs/?Bagu6o),[[3]](https://www.zotero.org/google-docs/?fJ0PwB), [4], [[5]](https://www.zotero.org/google-docs/?yg1vlT),[[6]](https://www.zotero.org/google-docs/?Kc4ZAK), only contains positive DTIs retrieved from different sources like KEGG BRITE[[7]](https://www.zotero.org/google-docs/?UP3vJq), DrugBank[[8]](https://www.zotero.org/google-docs/?a6lk4F), SuperTarget[[9]](https://www.zotero.org/google-docs/?rZPHVm), and BRENDA [[10]](https://www.zotero.org/google-docs/?EB5Yqj)databases. The chemical structures of drugs were obtained from the KEGG LIGAND database [[7]](https://www.zotero.org/google-docs/?UP3vJq) and the drug similarities were calculated[[11]](https://www.zotero.org/google-docs/?Ex3zYG). The target protein similarities were computed based on the Smith-Waterman similarity score [12] between amino acid sequences collected from the KEGG GENES database[[7]](https://www.zotero.org/google-docs/?UP3vJq). Target proteins are classified into four groups of enzymes, ion channels, G-protein-coupled receptors and nuclear receptors[13]. The dataset consists of 791 drugs and 989 targets connected through 5127 positive validated interactions. The class ratio shows an extreme class imbalance between positive and unknown/negative interactions[[2]](https://www.zotero.org/google-docs/?Bagu6o), [[3]](https://www.zotero.org/google-docs/?fJ0PwB). The entire dataset is publicly available at<http://web.kuicr.kyoto-u.ac.jp/supp/yoshi/drugtarget/>.

ChEMBL is an open bioactivity repository that advances the research in computational drug discovery[14],[[15]](https://www.zotero.org/google-docs/?K1xDtB)[)](https://www.zotero.org/google-docs/?0iwFQa). ChEMBL labelled DTIs as active (positive) or inactive (negative) and the ligand efficiencies of active interactions were calculated on pChEMBL value (negative log10 molar IC50, XC50, EC50, etc.)[[15]](https://www.zotero.org/google-docs/?K1xDtB). In this study, only “SINGLE PROTEIN”targets and “IC50” interactions were selected [16] and drugs without enough information ("SMILES'', “pChEMBL value”, and with fewer than 100 interactions) were excluded.

## **References**

[1. Ding H, Takigawa I, Mamitsuka H, Zhu S. Similarity-based machine learning methods for predicting drug–target interactions: a brief review. Brief Bioinform. 2014;15:734–47.](https://www.zotero.org/google-docs/?Y8pngI)

[2. Pliakos K, Vens C. Drug-target interaction prediction with tree-ensemble learning and output space reconstruction. BMC Bioinformatics. 2020;21:49.](https://www.zotero.org/google-docs/?Y8pngI)

[3. Thafar MA, Olayan RS, Ashoor H, Albaradei S, Bajic VB, Gao X, et al. DTiGEMS+: drug–target interaction prediction using graph embedding, graph mining, and similarity-based techniques. Journal of Cheminformatics. 2020;12:44.](https://www.zotero.org/google-docs/?Y8pngI)

[4. Bagherian M, Sabeti E, Wang K, Sartor MA, Nikolovska-Coleska Z, Najarian K. Machine learning approaches and databases for prediction of drug–target interaction: a survey paper. Briefings in Bioinformatics. 2021;22:247–69.](https://www.zotero.org/google-docs/?Y8pngI)

[5. Mohamed SK, Nováček V, Nounu A. Discovering Protein Drug Targets Using Knowledge Graph Embeddings. Bioinformatics. 2019;:btz600.](https://www.zotero.org/google-docs/?Y8pngI)

[6. Hao M, Bryant SH, Wang Y. Predicting drug-target interactions by dual-network integrated logistic matrix factorization. Sci Rep. 2017;7. doi:10.1038/srep40376.](https://www.zotero.org/google-docs/?Y8pngI)

[7. Kanehisa M, Goto S, Hattori M, Aoki-Kinoshita KF, Itoh M, Kawashima S, et al. From genomics to chemical genomics: new developments in KEGG. Nucleic Acids Research. 2006;34 suppl_1:D354–7.](https://www.zotero.org/google-docs/?Y8pngI)

[8. Wishart DS, Knox C, Guo AC, Cheng D, Shrivastava S, Tzur D, et al. DrugBank: a knowledgebase for drugs, drug actions and drug targets. Nucleic Acids Research. 2008;36 suppl_1:D901–6.](https://www.zotero.org/google-docs/?Y8pngI)

[9. Günther S, Kuhn M, Dunkel M, Campillos M, Senger C, Petsalaki E, et al. SuperTarget and Matador: resources for exploring drug-target relationships. Nucleic Acids Research. 2008;36 suppl_1:D919–22.](https://www.zotero.org/google-docs/?Y8pngI)

[10. Schomburg I, Chang A, Ebeling C, Gremse M, Heldt C, Huhn G, et al. BRENDA, the enzyme database: updates and major new developments. Nucleic Acids Research. 2004;32 suppl_1:D431–3.](https://www.zotero.org/google-docs/?Y8pngI)

[11. Hattori M, Okuno Y, Goto S, Kanehisa M. Development of a chemical structure comparison method for integrated analysis of chemical and genomic information in the metabolic pathways. J Am Chem Soc. 2003;125:11853–65.](https://www.zotero.org/google-docs/?Y8pngI)

[12. Smith TF, Waterman MS. Identification of common molecular subsequences. Journal of Molecular Biology. 1981;147:195–7.](https://www.zotero.org/google-docs/?Y8pngI)

[13. Yamanishi Y, Araki M, Gutteridge A, Honda W, Kanehisa M. Prediction of drug–target interaction networks from the integration of chemical and genomic spaces. Bioinformatics. 2008;24:i232–40.](https://www.zotero.org/google-docs/?Y8pngI)

[14. Lenselink EB, ten Dijke N, Bongers B, Papadatos G, van Vlijmen HWT, Kowalczyk W, et al. Beyond the hype: deep neural networks outperform established methods using a ChEMBL bioactivity benchmark set. Journal of Cheminformatics. 2017;9:45.](https://www.zotero.org/google-docs/?Y8pngI)

[15. Bento AP, Gaulton A, Hersey A, Bellis LJ, Chambers J, Davies M, et al. The ChEMBL bioactivity database: an update. Nucleic Acids Research. 2014;42:D1083–90.](https://www.zotero.org/google-docs/?Y8pngI)

[16. Cardoso-Silva J, Papageorgiou LG, Tsoka S. Network-based piecewise linear regression for QSAR modelling. J Comput Aided Mol Des. 2019;33:831–44.](https://www.zotero.org/google-docs/?Y8pngI)
