# Supplementary figures and images for: Novel drug-target interactions via link prediction and network embedding
Source: BMC Bioinformatics. 2022 Apr 4;23:121. doi: 10.1186/s12859-022-04650-w (PMC8978405; doi:10.1186/s12859-022-04650-w)

(a)

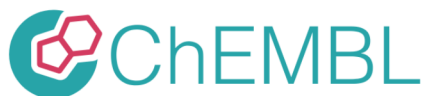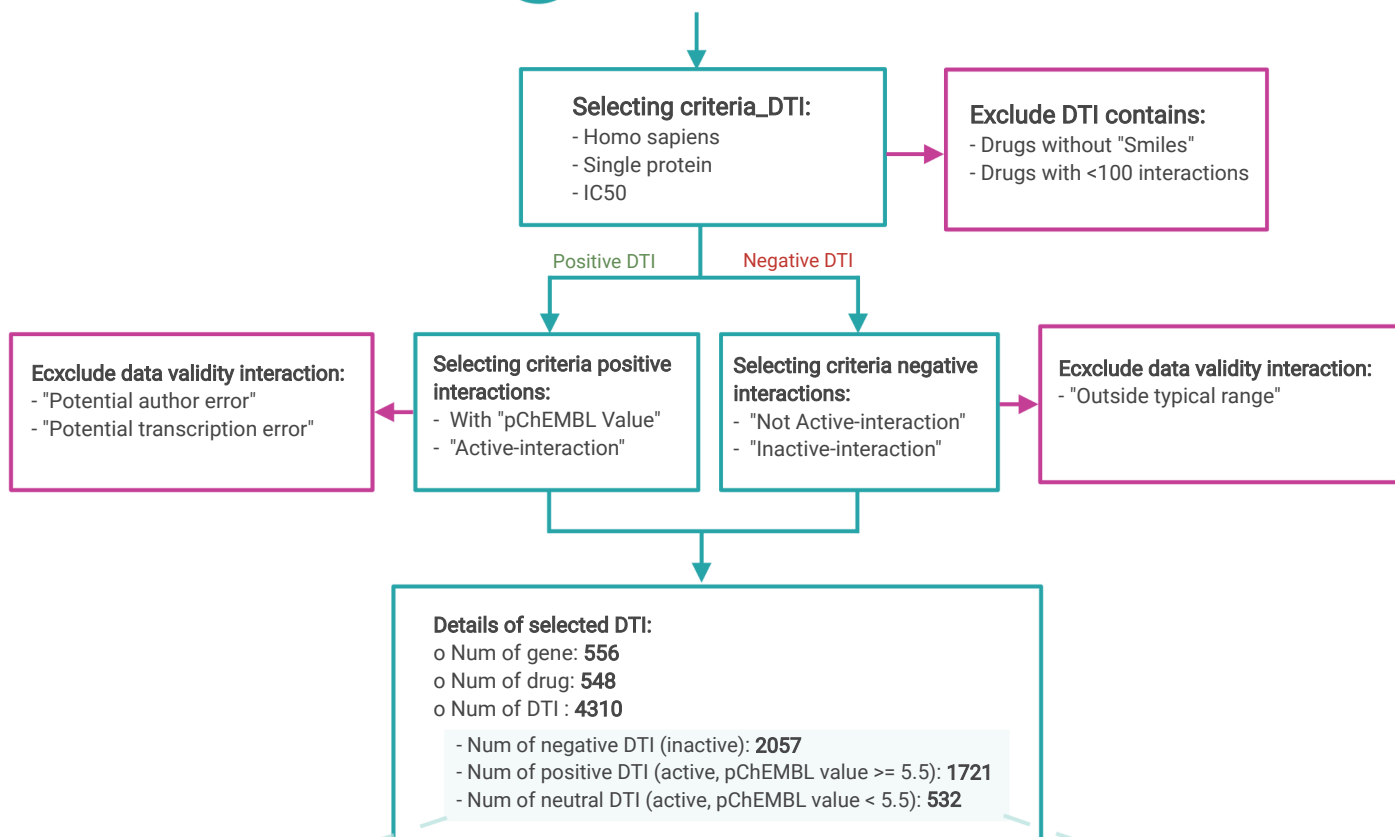

(b)

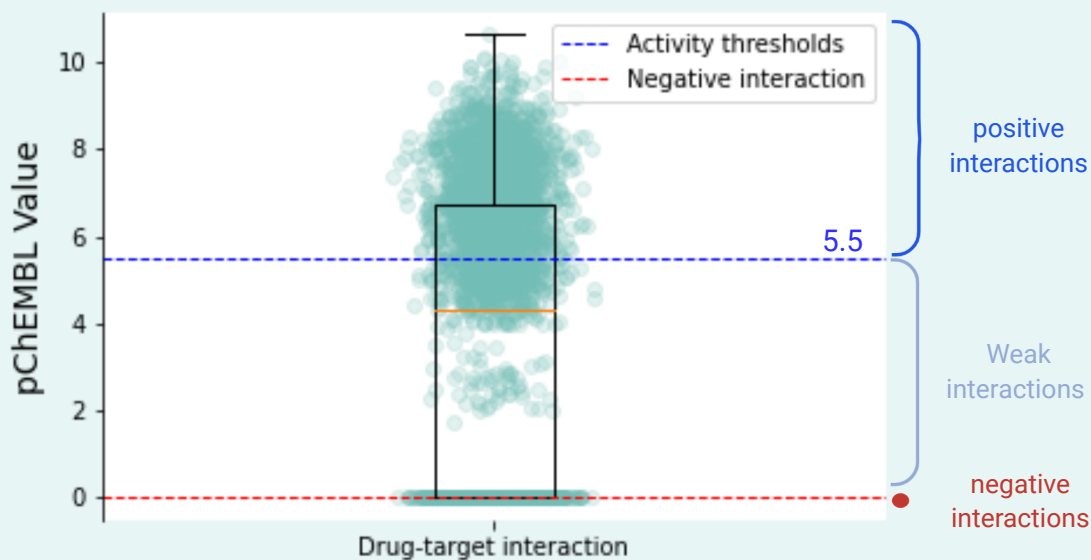

Supplement: Supplementary file 3 — Additional file 3: Figure S1. Data collection procedure. (a) Workflow to collect data from the ChEMBL database, (b) Scatter plot of the pChEMBL value of collected DTIs on a boxplot. [file 12859_2022_4650_MOESM3_ESM.pdf]

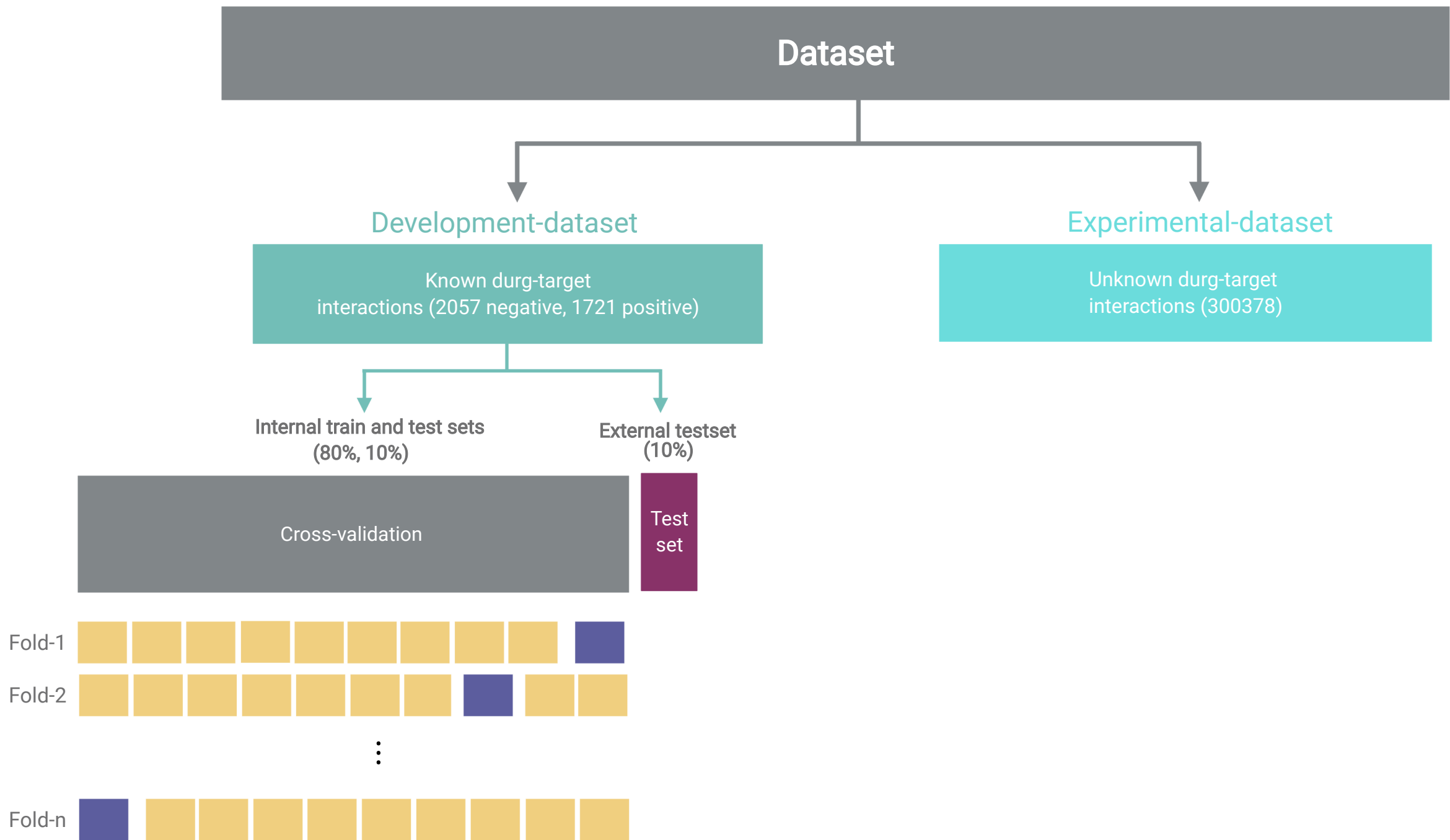

Supplement: Supplementary file 4 — Additional file 4: Figure S2. Datasets and data splitting. Details of data types and data splitting in cross-validation. [file 12859_2022_4650_MOESM4_ESM.pdf]

(a) Golden standard dataset

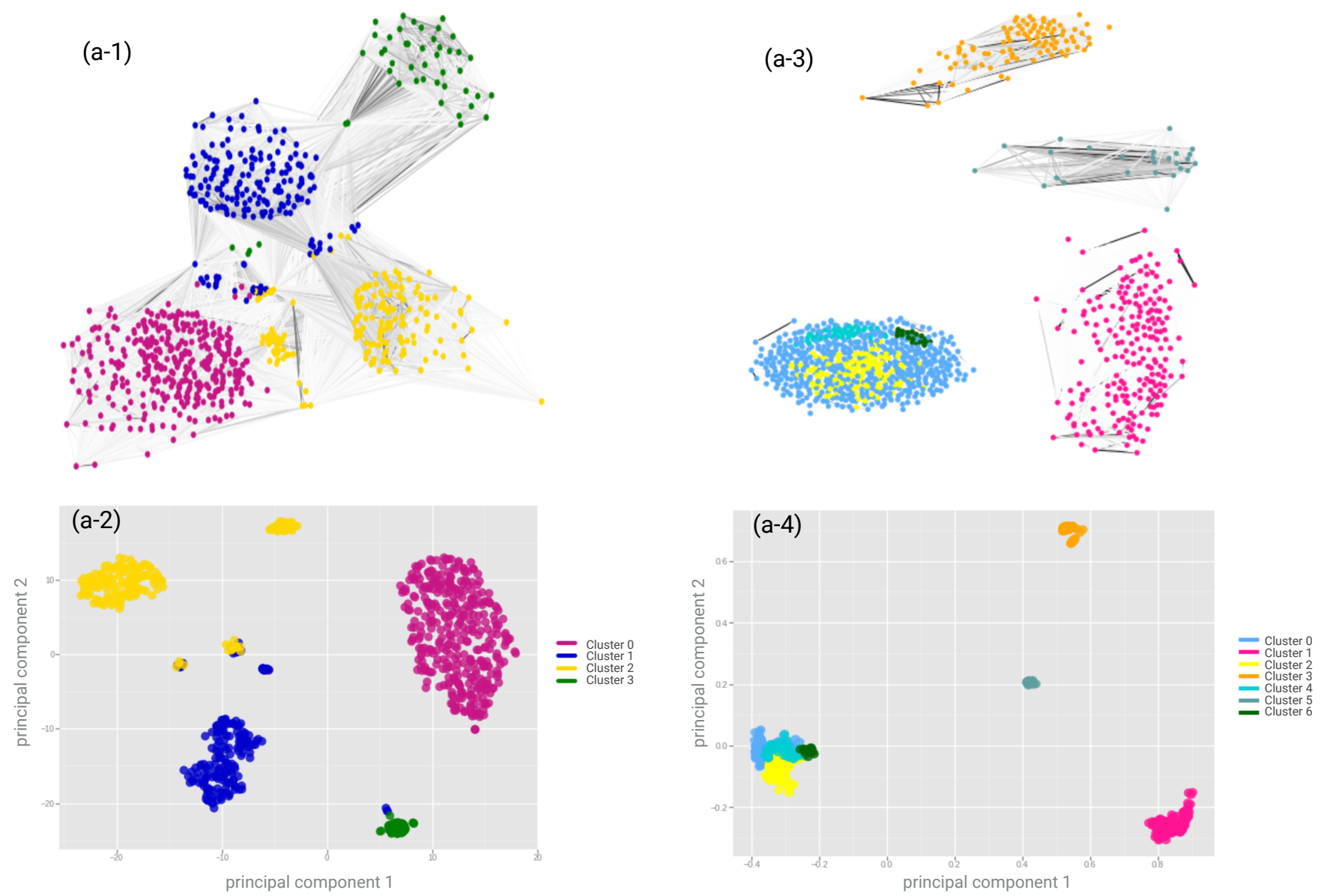

(b) ChEMBL dataset

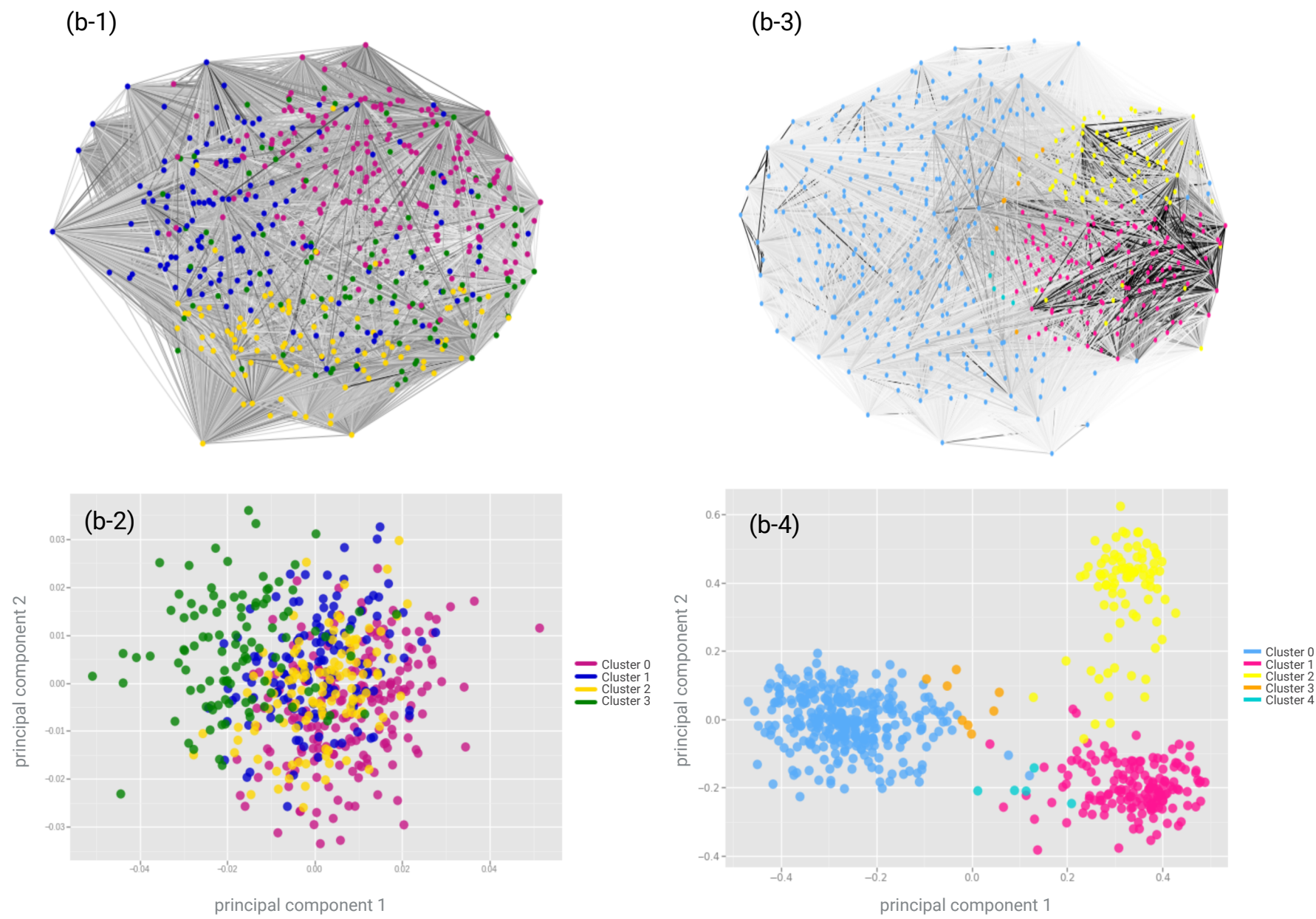

Supplement: Supplementary file 5 — Additional file 5: Figure S3. Networks of drug and protein similarities. Topological representation of the DDS and PPS networks and PCA of drugs and targets based on embedded vectors. [file 12859_2022_4650_MOESM5_ESM.pdf]

(a) Golden standard dataset

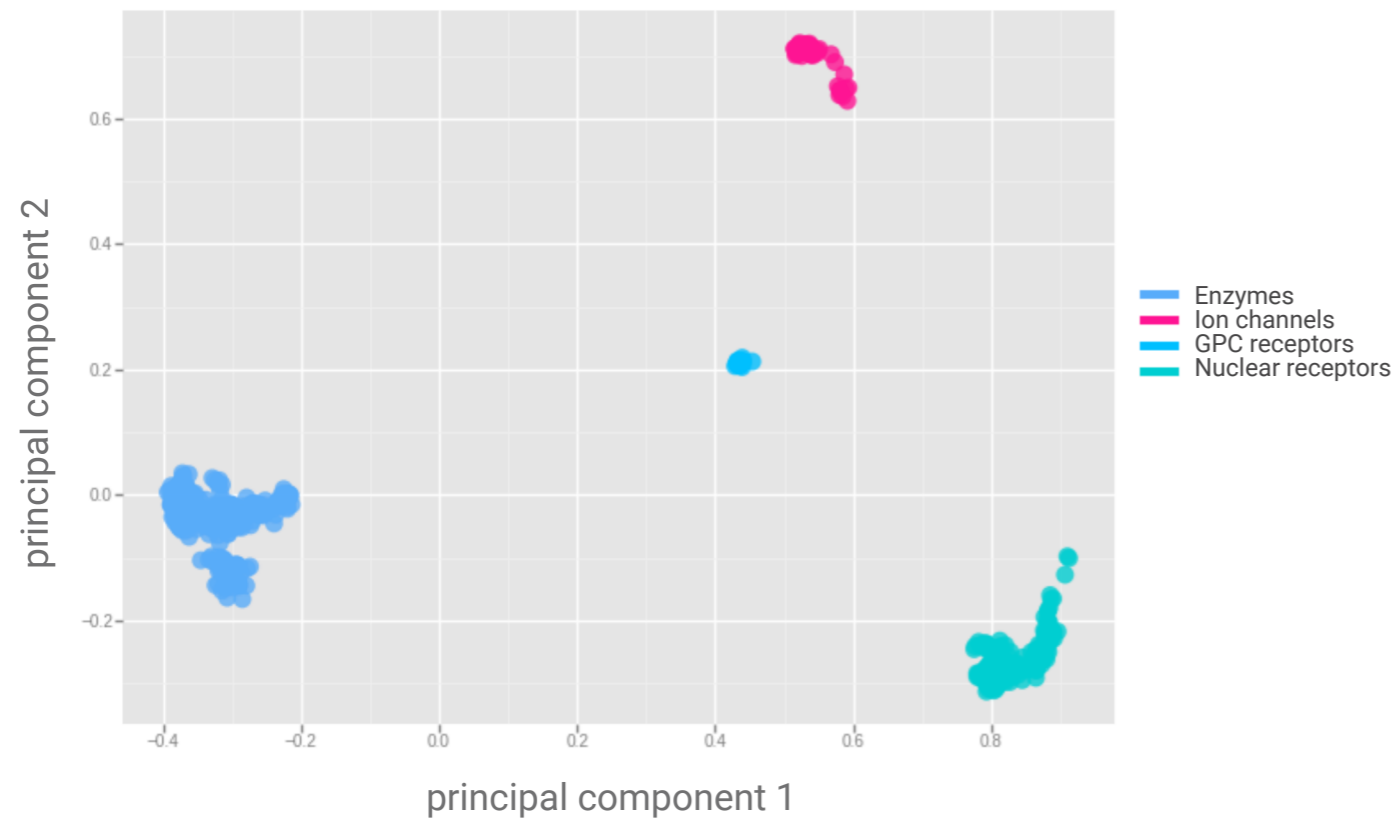

(b) ChEMBL dataset

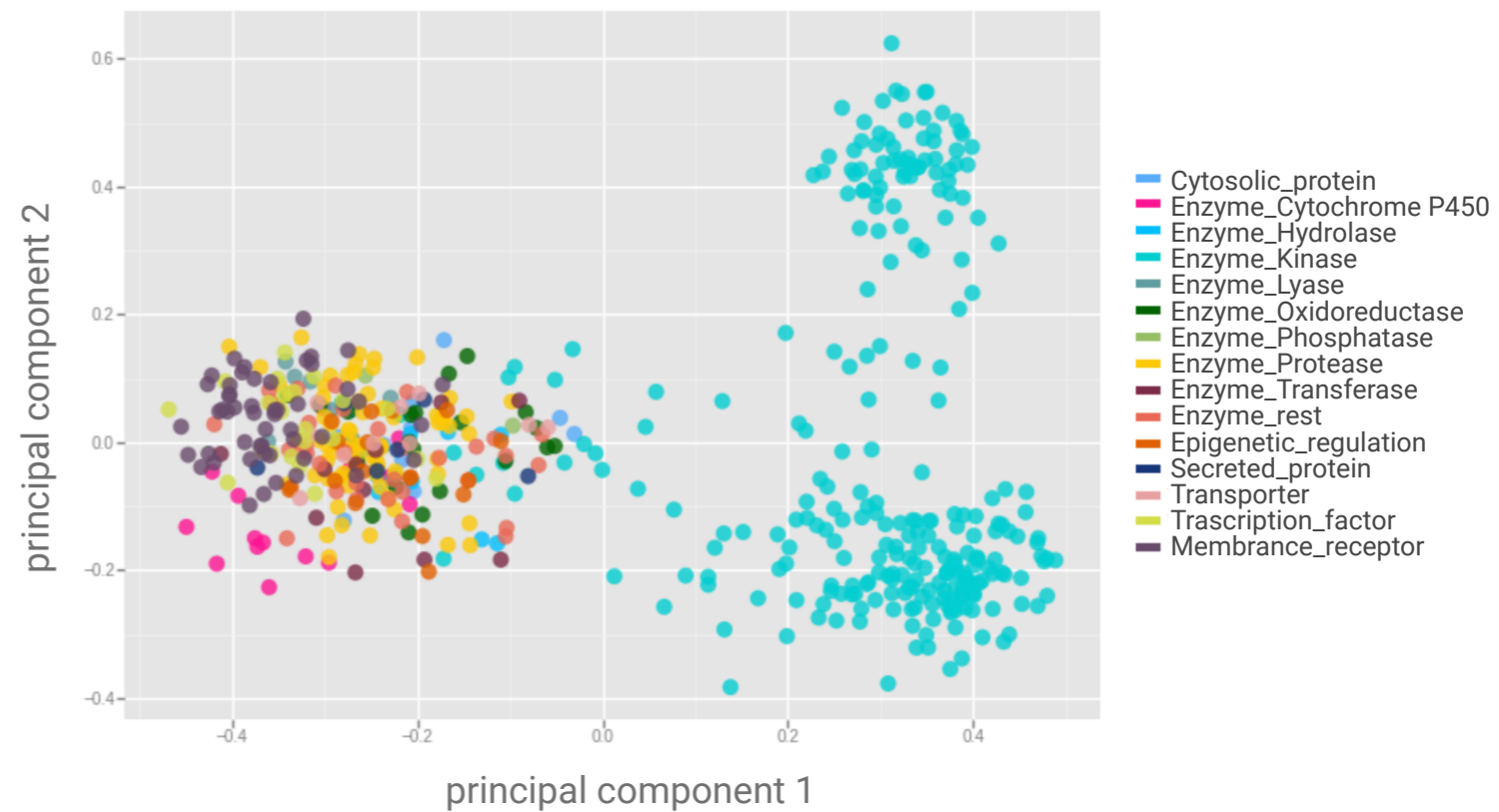

Supplement: Supplementary file 6 — Additional file 6: Figure S4. PCA plots. PCA of embedded vectors of proteins coloured according to protein type. [file 12859_2022_4650_MOESM6_ESM.pdf]

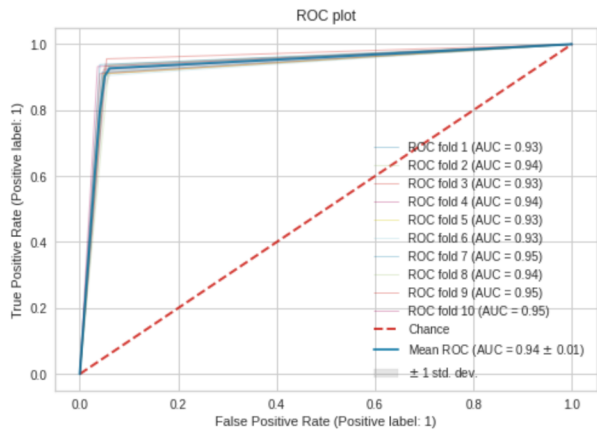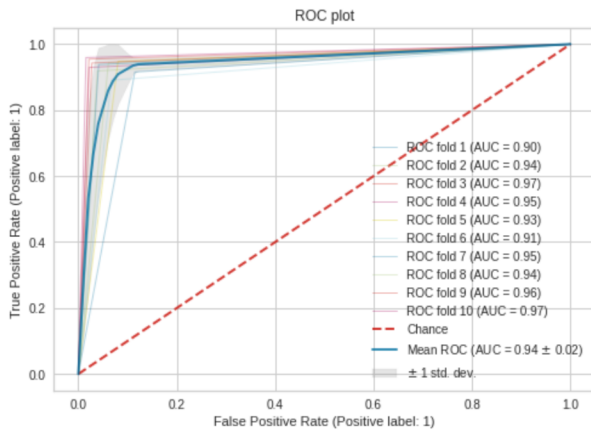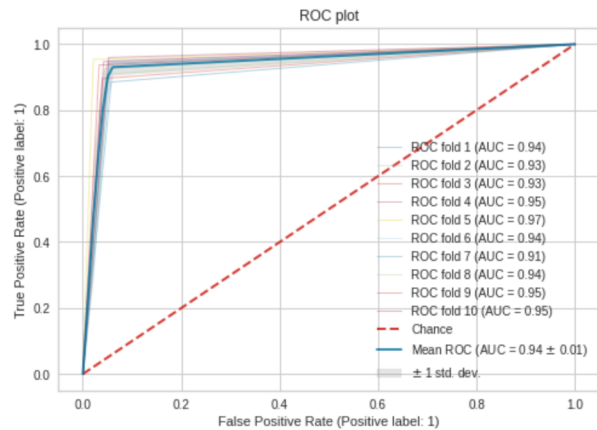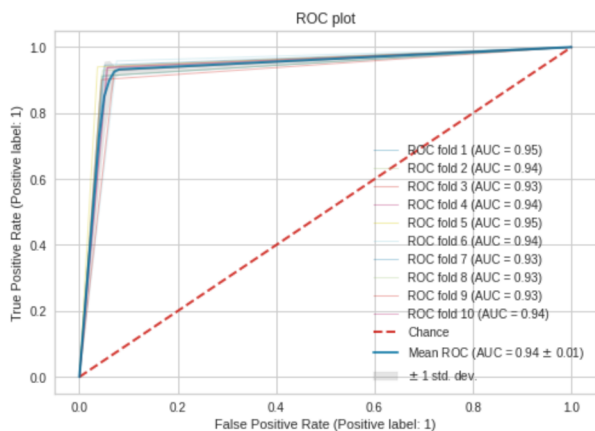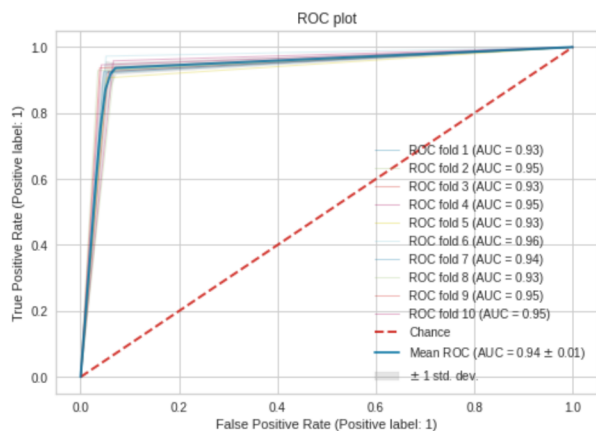

Supplement: Supplementary file 7 — Additional file 7: Figure S5. ROC plots over the ten-fold five times cross-validation. [file 12859_2022_4650_MOESM7_ESM.pdf]

(a-1)

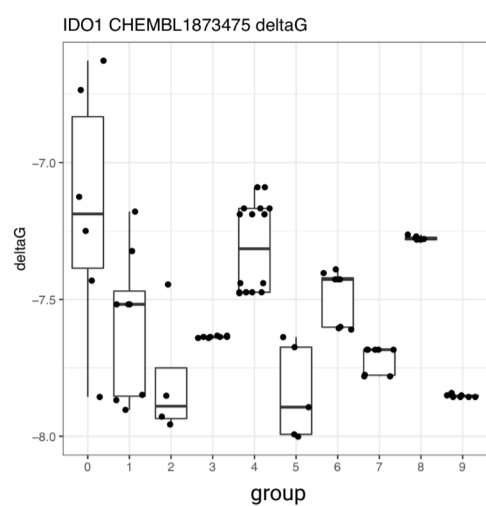

(a-2)

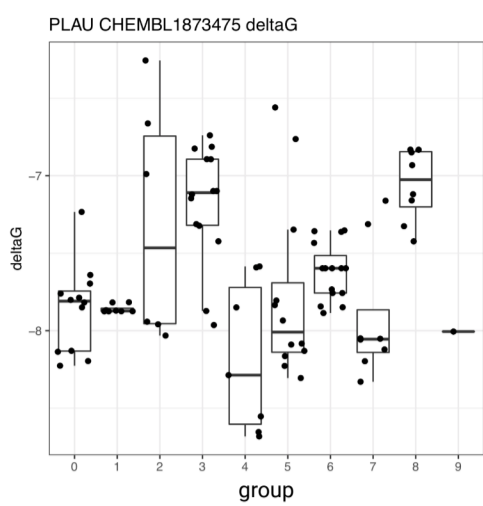

(a-3)

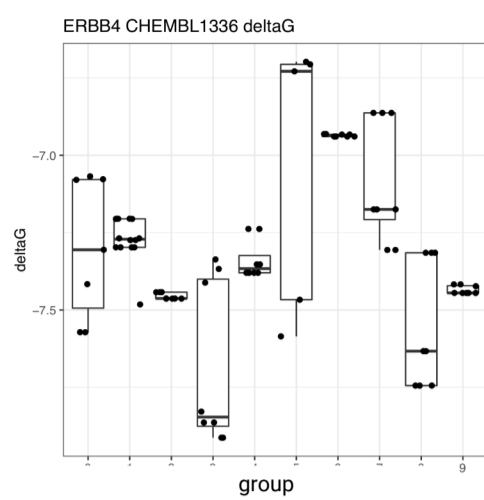

(a-4)

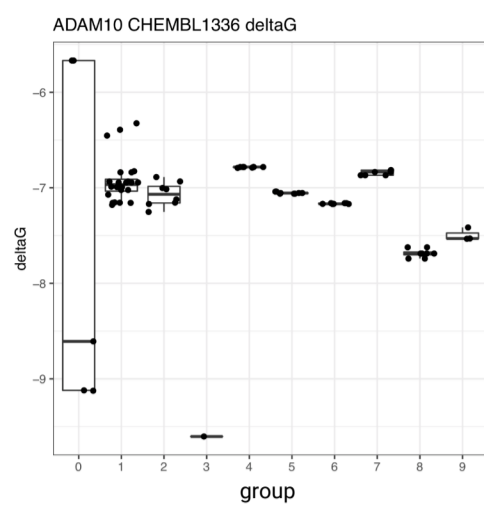

(a-5)

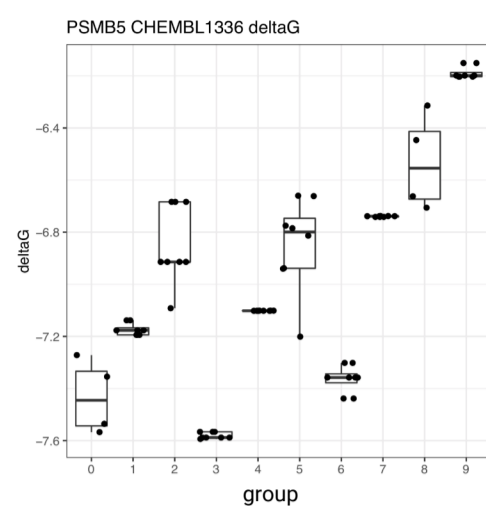

(b-1)

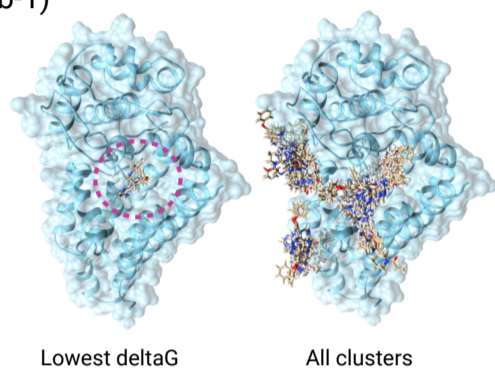

(b-2)

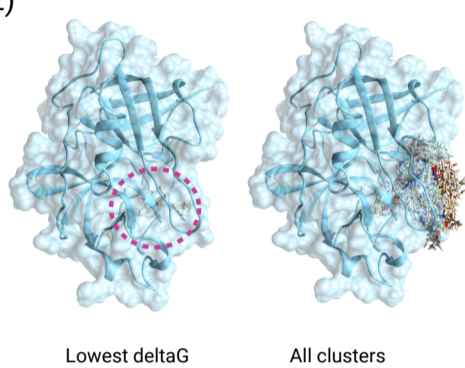

(b-3)

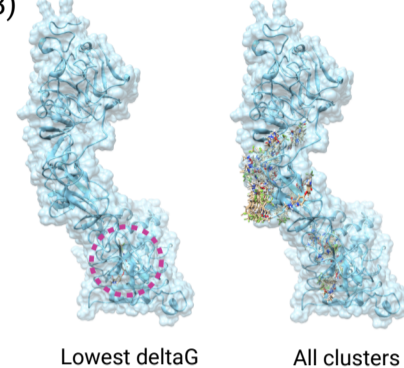

(b-4)

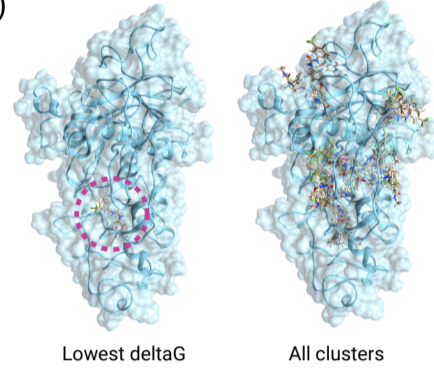

(b-5)

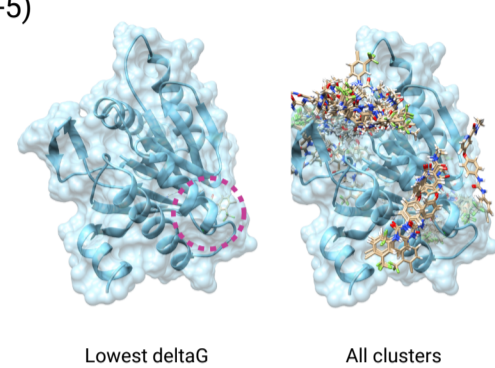

(a-6)

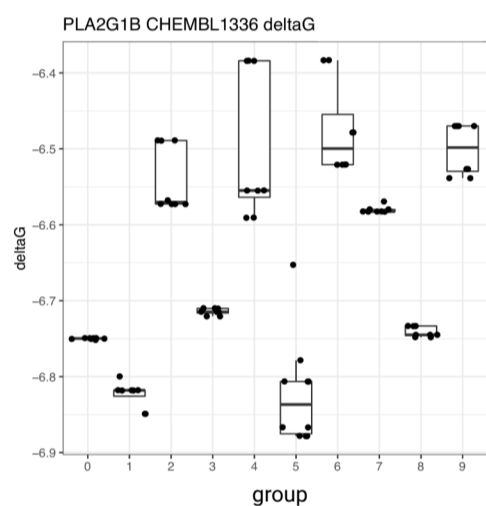

(a-7)

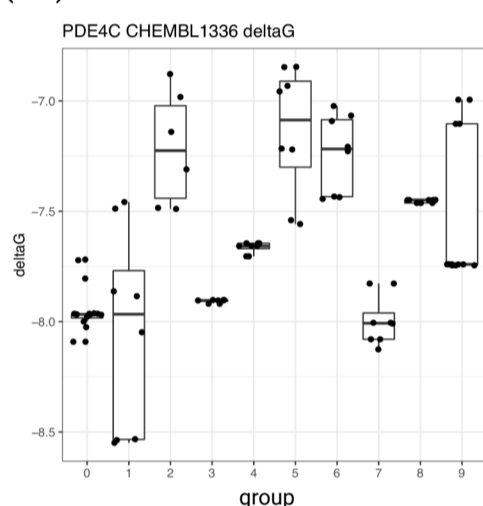

(a-8)

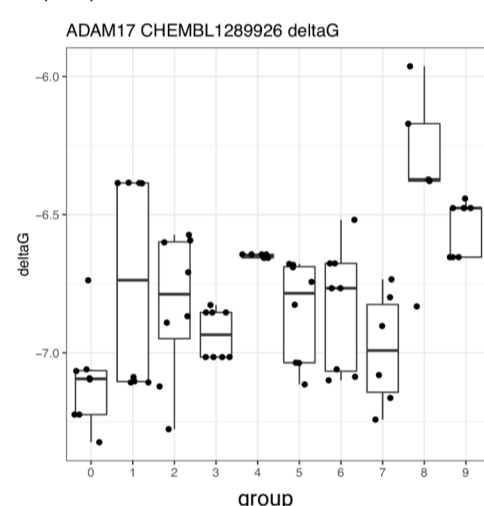

(a-9)

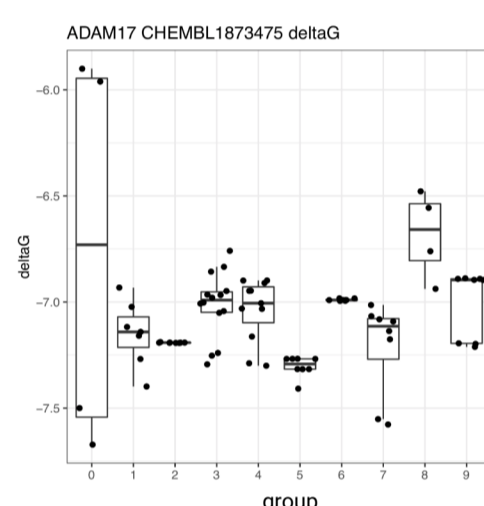

(a-10)

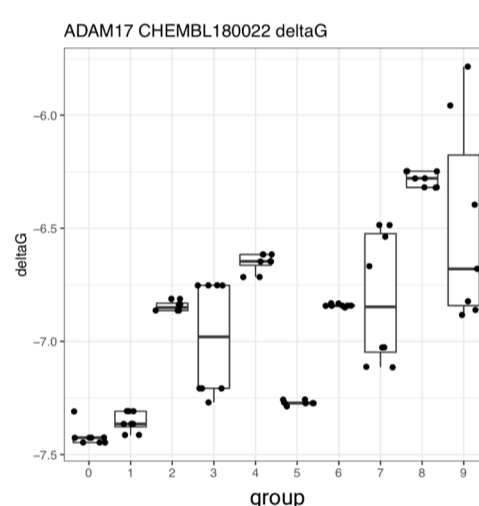

(b-6)

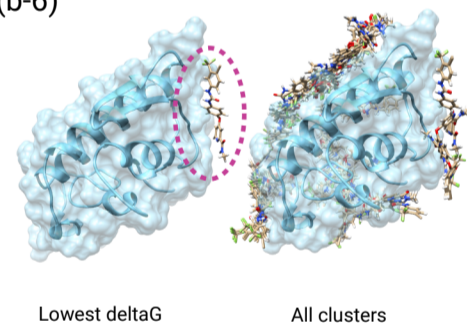

(b-7)

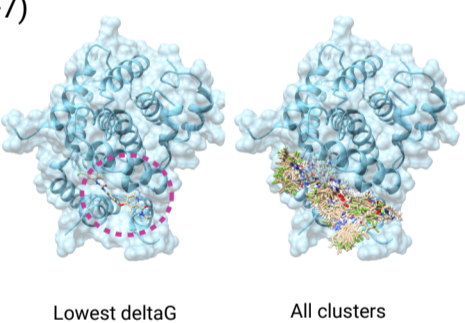

(b-8)

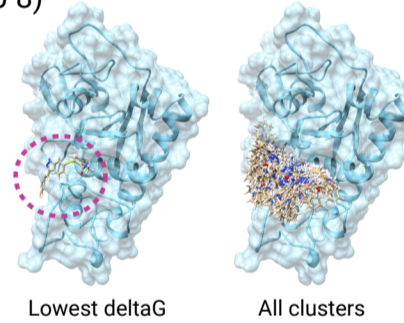

(b-9)

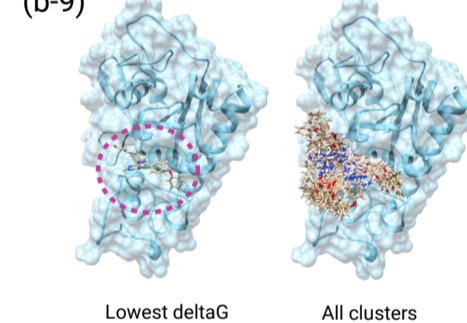

(b-10)

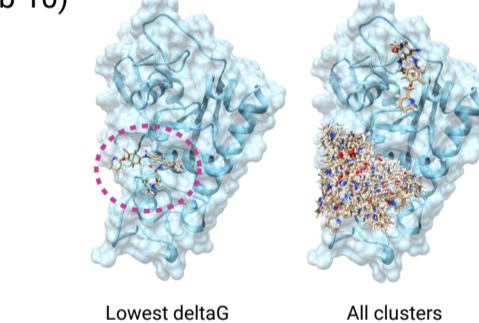

(a-11)

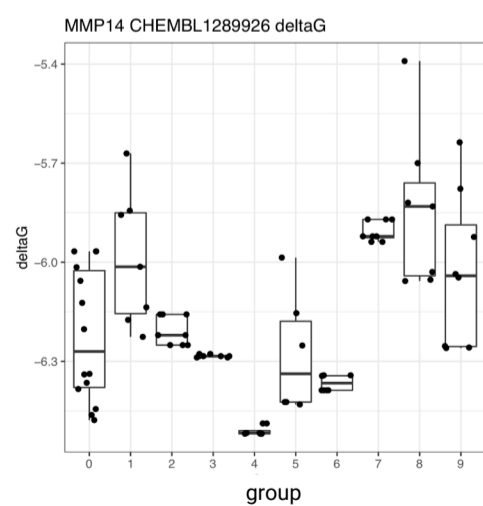

(a-12)

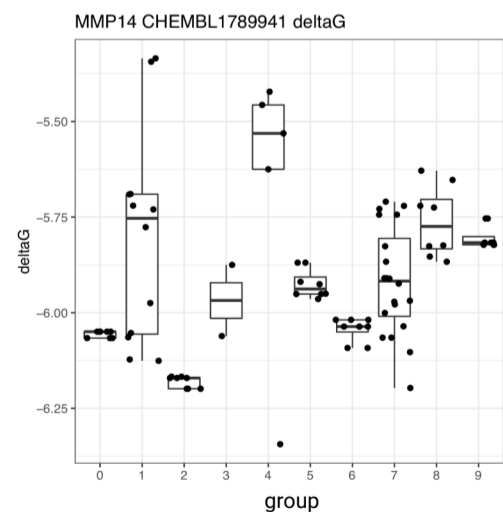

(a-13)

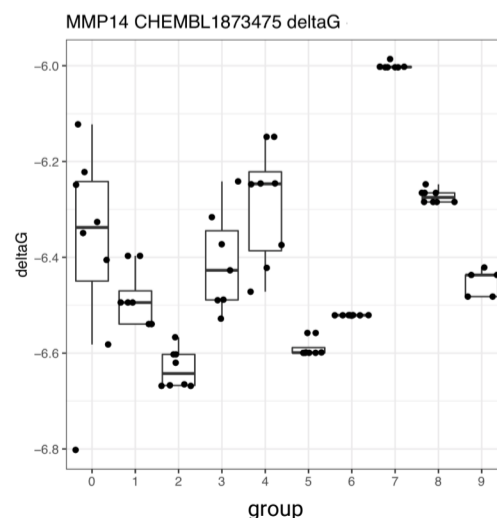

(b-11)

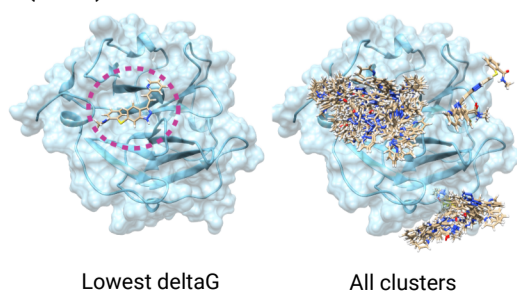

(b-12)

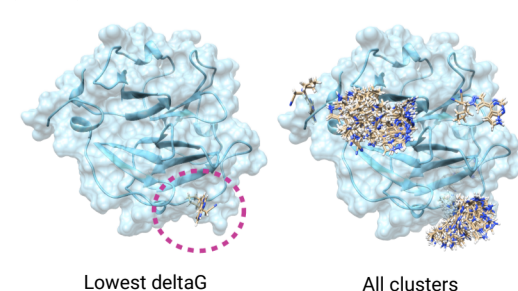

(b-13)

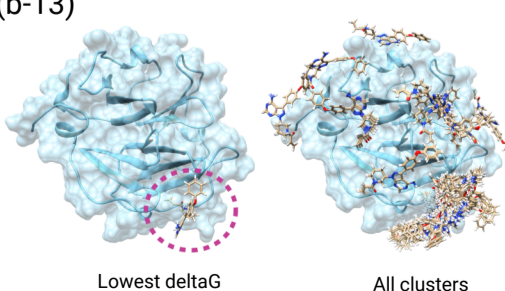

Supplement: Supplementary file 8 — Additional file 8: Figure S6. DTIs through docking. Molecular docking performed via SwissDock for novel predicted DTIs. (a) deltaG for the first 10 groups of molecules clustered by conformers similarity. (b) Binding locations with the lowest deltaG and all groups of conformers. [file 12859_2022_4650_MOESM8_ESM.pdf]

(a)

DDS between compounds predicted for ADAM17

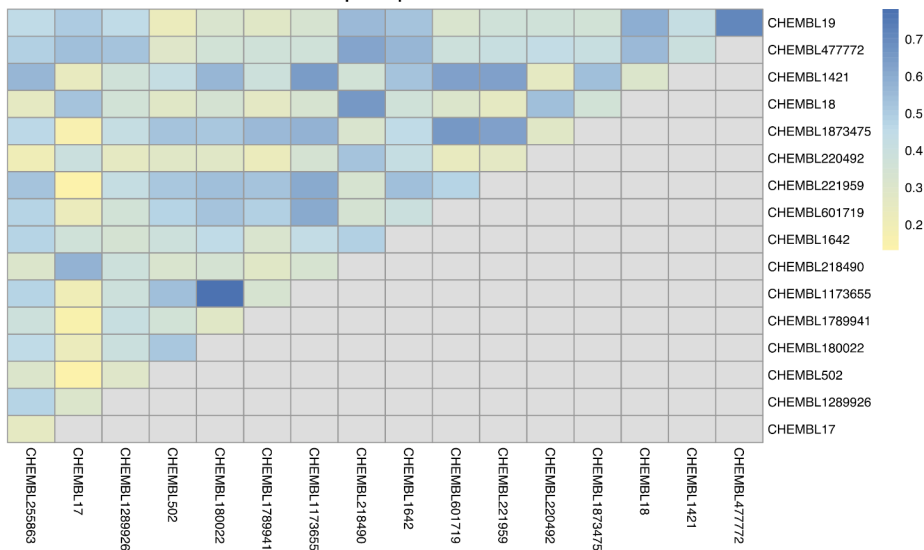

(b)

DDS between compounds predicted for MMP14

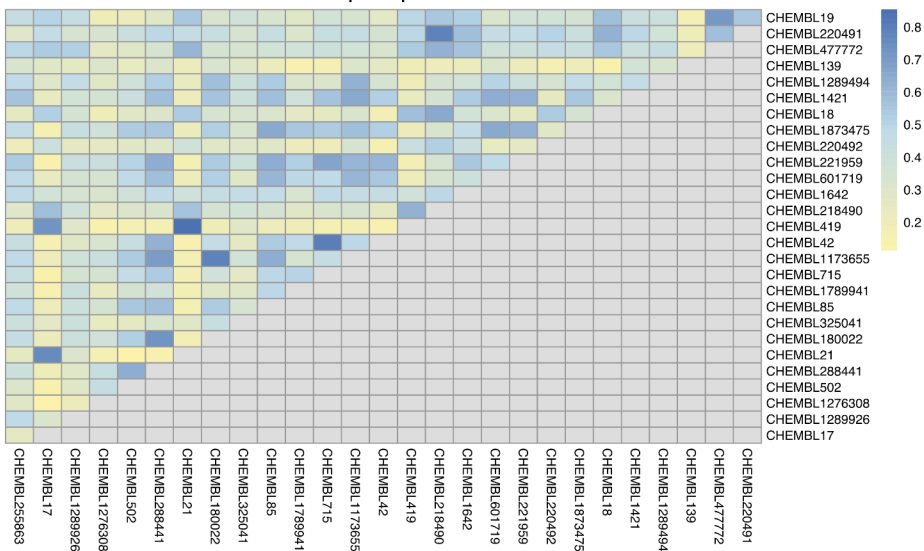

Supplement: Supplementary file 9 — Additional file 9: Figure S7. Drug similarity heatmaps. Drug-drug similarity for drugs predicted to interact with (a) ADAM17 and (b) MMP14. [file 12859_2022_4650_MOESM9_ESM.pdf]
